# COVID-19: Development and implementation of a video-conference-based educational concept to improve the hygiene skills of health and nursing professionals in the Republic of Kosovo

**DOI:** 10.3205/dgkh000412

**Published:** 2022-06-03

**Authors:** Leonie Macht, Dieter Worlitzsch, Naime Braijoshri, Petrit Bequiri, Jacqueline Zudock, Max Zilezinski, Dietrich Stoevesandt, Jamie Smith, Sebastian Hofstetter

**Affiliations:** 1 Universitätsklinikum Halle (Saale), Stabsstelle Krankenhaushygiene, Halle (Saale), Germany; 2 Kolegji Heimerer, Pristina, Republic of Kosovo; 3 Kolegji Heimerer, Lagja Kalabri, Pristina, Republic of Kosovo; 4 Martin-Luther-Universität Halle-Wittenberg, Dorothea Erxleben Lernzentrum, SkillsLab, Halle (Saale), Germany; 5 Martin-Luther-Universität Halle-Wittenberg, AG Versorgungsforschung, Halle (Saale), Germany; 6 Charité-Universitätsmedizin Berlin, Institut für Klinische Pflegewissenschaft, Berlin, Germany

**Keywords:** Covid-19-pandemic, hygiene, advanced training, international educational exchange, health care professionals

## Abstract

**Introduction:**

The COVID-19 pandemic caught the health care systems of all countries unprepared. For the further education of healthcare personnel in the Republic of Kosovo, it became necessary to implement a concept for practical training in hygienic working. A video-conference–based educational concept to bridge the physical distance between Germany and Kosovo enabled the rapid, theoretical and practical transfer of knowledge.

**Methods:**

Current evidence on COVID-19 and Standard Operation Procedures (SOP) were researched. Healthcare staff in Pristina were advised and trained in ten online sessions on hygienic working under pandemic conditions via the “Logitech Rally for DFNconf” video conferencing system. The seminars were interpreted consecutively (Albanian). The Situational Judgement Test (HygiKo-SJT) should describe changes in participants’ hygiene knowledge.

**Results:**

A total of 25 people were trained in hygiene-related knowledge in terms of basic and specific hygiene for COVID-19. The weekly training sessions made it possible to address questions and subsequently provide practical knowledge. The HygiKo-SJT did not show a generalizable, measurable improvement in hygiene competence.

**Conclusion:**

Participants benefited from the concept and rapid implementation of a live video-conference–based seminar on “Hygiene under Pandemic Conditions”. The positive experience in terms of guidance, advice and training provides the basis for implementing a dedicated “Hygiene” module in Kosovo

## Zusammenfassung

**Zielsetzung:**

Die COVID-19-Pandemie hat die Gesundheitssysteme aller Länder unvorbereitet getroffen. Für die Weiterbildung des Gesundheitspersonals in der Republik Kosovo ergab sich die Notwendigkeit, ein Konzept zur praxisnahen Schulung hygienegerechten Arbeitens zu implementieren. Durch ein videokonferenzbasiertes Edukationskonzept zur Überbrückung der räumlichen Distanz zwischen Deutschland und dem Kosovo war die schnelle, theoretische und praxisnahe Wissensvermittlung möglich.

**Methode:**

Aktuelle Evidenz zum Thema COVID-19 sowie die Standard Operation Procedures (SOP) wurden recherchiert sowie das Gesundheitspersonal in Pristina via Video-Konferenzsystem „Logitech Rally für DFNconf“ in zehn Online-Sitzungen zu hygienischem Arbeiten unter Pandemiebedingungen beraten und geschult. Die Seminare wurden konsekutiv gedolmetscht (albanisch). Der Situational Judgement Test (HygiKo-SJT) sollte Veränderung im Hygienewissen der Teilnehmenden beschreiben.

**Ergebnisse:**

Insgesamt bildeten sich 25 Personen zu hygienerelevantem Wissen im Bereich Basis- und spezieller Hygiene zu COVID-19 fort. Durch die wöchentlichen Schulungstermine war es möglich, Fragen aufzugreifen und darauf folgend praxisnahes Wissen bereit zu stellen. Eine generalisierbare, messbare Verbesserung der Hygienekompetenz zeigte der HygiKo-SJT nicht.

**Schlussfolgerung:**

Die Teilnehmenden profitierten vom Konzept und der schnellen Umsetzung eines videokonferenzbasierten Live-Seminars „Hygiene unter Pandemiebedingungen“. Die positiven Erfahrungen hinsichtlich Anleitung, Beratung und Schulung liefern die Grundlagen für die Implementierung eines eigenen Moduls „Hygiene“ im Kosovo.

## Introduction

Since its outbreak at the beginning of 2020 [1], the COVID-19 pandemic has burdened the health systems of all countries. The rapid increase in hospitalized patients during the COVID-19 pandemic posed a significant problem worldwide [2]. In order to enable high-quality patient care and ensure patient safety, it is necessary for all healthcare professionals working in clinics to have in-depth knowledge of infection control and hygiene measures. Against this background, at the beginning of the pandemic, the idea of " making innovative projects from different locations visible " arose [3]. A digital education programme was planned to deliver relevant hygiene knowledge from Germany to the Republic of Kosovo using a video conference system. The potential of digital technologies to deliver high-quality education was shown in preliminary work [3], [4], [5]. The Dorothea Erxleben Learning Center/SkillsLab of the Medical Faculty of the Martin Luther University Halle-Wittenberg has extensive previous experience in conventional and digital teaching [4], [5], [6], [7], [8], [9]. 

When the project was planned, the Republic of Kosovo was badly affected by Covid-19 [10]. The development and implementation of a video-conference concept to introduce, advise and train nurses and medical staff in the Republic of Kosovo was a reaction to the particular requirements for specific infection control measures for the COVID-19 pandemic. The funds, resources and skills required to develop an educational concept are limited in the Western Balkans, because health infrastructure, access, available funds and governance differ between countries with low and high gross domestic products (GDP). The Republic of Kosovo is one of the countries in Europe with the lowest share of its GDP made up of health expenditures, with per capita health expenditures being almost 12 times lower than the pan-European level [11], [12], [13]. For this reason, cooperation between Martin Luther University and the Kolegji Heimerer in Pristina was established. Kolegji Heimerer is a German-Kosovan university of applied sciences that offers higher education in health and nursing sciences. The cooperation aimed to make evidence-based, hygiene-relevant knowledge quickly and directly available in the Republic of Kosovo. Digital contact options open up a new perspective for knowledge transfer and are a turning point in the field of e-health [14].

### Problem and question

Hospital hygiene measures are a fundamental part of COVID-19 infection prevention. In the worst case, a lack of knowledge in this area can endanger patients and employees [15]. In the Republic of Kosovo, hospital hygiene content is not yet a regular part of the curriculum both in nursing and sometimes also in human medicine [16], [17]. As a result, the transfer of hygiene-related knowledge and its application is of great importance, both scientifically and practically, regardless of pandemic events. Using the example of the Republic of Kosovo, Raka et al. [16] state that a healthcare system with insufficient budgets and an insufficient number of health professionals trained in infection prevention are reasons for a high rate of nosocomial infections. For the Republic of Kosovo, the broad availability of hospital hygiene knowledge is currently relevant for managing COVID-19. When the project started, the Republic of Kosovo was already severely affected by the Covid-19 pandemic, with around 14,500 cases in a population of around 1.8 million (World Health Organization Coronavirus Dashboard, as of 09/2020). This raised the question of how hygiene-relevant knowledge can be made available to nursing and medical staff in Kosovo promptly, without barriers, and how the success of knowledge transfer can be assessed.

### Objective

The first objective of this project was to collect and translate available evidence on infection control and relevant standard hygiene operating procedures (SOP). and make it available to colleagues. The second objective was to develop an educational concept for digital hygiene training to offer nursing and medical staff in the Republic of Kosovo an easy-to-implement and feasible option for knowledge transfer. Due to the acute situation of the COVID-19 pandemic, a swift response was required. Therefore, a third project objective was a short-term and sustainable transfer of knowledge on hygiene measures using a video conference system. 

## Project description

Knowledge transfer is understood as the targeted transfer of “know-how” from a knowledge sender to a knowledge receiver for problem-solving, and includes both the contents to be transferred and all activities necessary for the transfer [18]. In order to implement an educational concept with suitable content quality, we collaborated with a local partner, the Training Academy for Health and Nursing Professions Kolegji Heimerer in Pristina. Together, a two-phase programme was developed to enable the transfer of hygiene-related knowledge. 

1^st^ phase (5 months from September 2020 to January 2021): preliminary research, preparation and summary of relevant evidence and current literature from the highly dynamic COVID-19 hygiene research as well as the Standard Operation Procedures (SOP) of the University Hospital Halle (Saale). 

**Figure 1:**
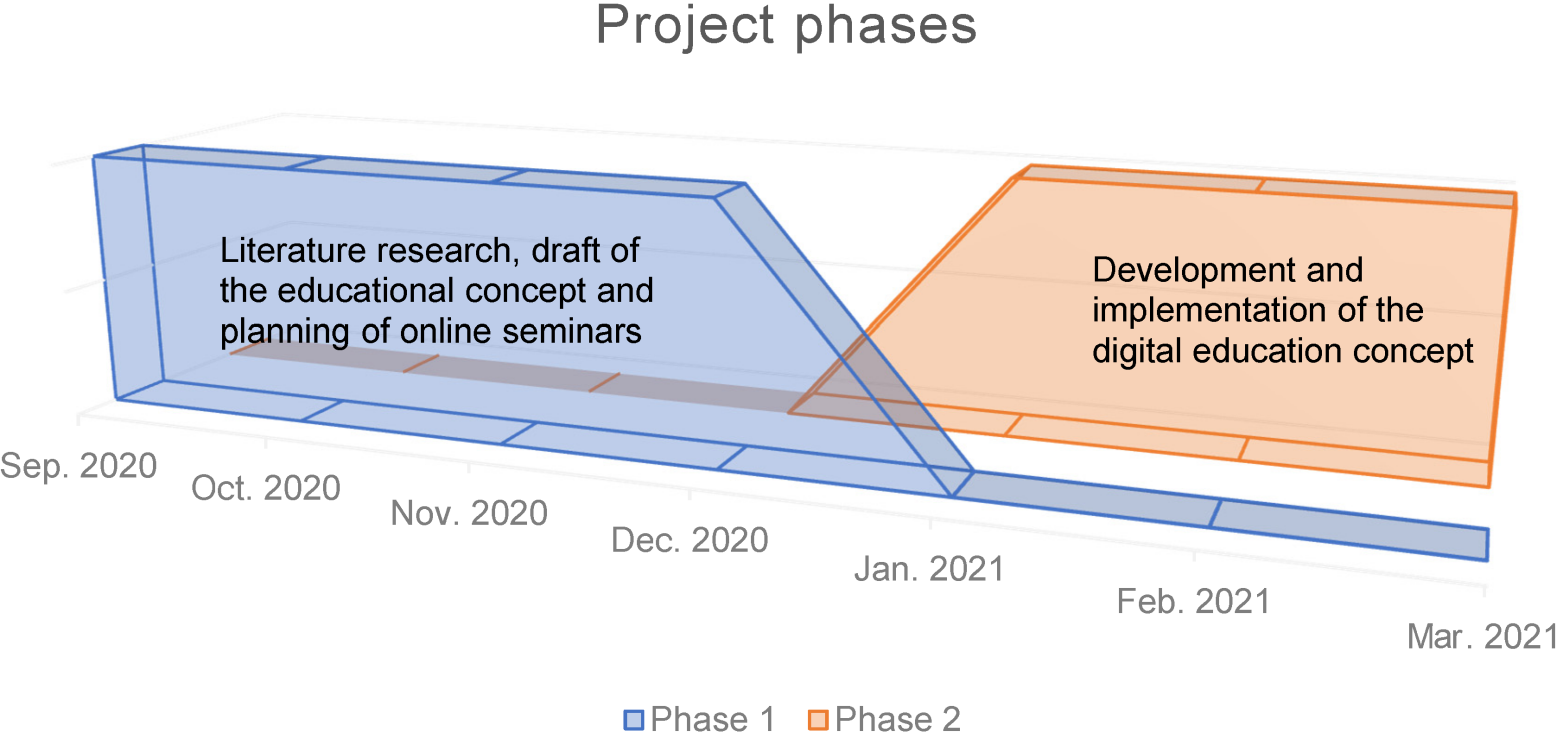
Schematic time course of the overall project

2^nd^ phase (3 months from December 2020–March 2021): development and implementation of a digital education programme using a video conferencing system (see Figure 1 (Fig. 1)). 

In the first phase, the results of a structured, topic-related literature research and a compilation of the available Standard Operation Procederes (SOP) of the University Hospital Halle (Saale) were made available to the Republic of Kosovo. Supported by German-speaking colleagues of the Kolegji Heimerer in Pristina, the SOPs were translated from German into Albanian. This enabled knowledge of basic hygiene measures and specific processes to be accessible to healthcare staff in preparation for COVID-19. The educational concept was based on moving from general topics of infection control and hygiene to more specific ones related to COVID-19.

Phase 1 [19] concluded with a needs analysis and the production of needs-based learning objectives to guide Phase 2. In Phase 2, 10 video conference-based 90-minute sessions spread over five weeks were developed. These, like the curriculum objectives, follow Sackett’s five steps of evidence-based medicine [20]. They reflect and integrate a systematic and logical sequence of actions fundamentally associated with the development and dissemination of evidence-based medicine or evidence-based health care. The online seminars were interpreted consecutivly into Albanian. Consecutive interpretation means that the translator speaks after the source-language speaker has spoken. For this purpose, the spoken text was divided into segments and translated from the source language into the target language by the interpreter during pauses in the speaker’s speech or at the end of defined text passages [21], [22].

During the individual online sessions, participants were able to ask questions about specific problem situations, which were either discussed directly or answered by additional, specific information materials as needed. For some situations, practical exercises were demonstrated (e.g., correct performance of hygienic hand disinfection and verification using fluorescent disinfectant and UV blacklight, correct donning and doffing of personal protective equipment (PPE), protective measures in special situations such as during resuscitation). Knowledge transfer from the online training was evaluated using a Situational Judgement Test (SJT) [23] at the beginning and end of the seminar series. The HygiKo-SJT uses picture vignettes to describe everyday hygiene-relevant situations in the clinical setting (e.g., behaviour during examination situations, patient transport, in the operating room) and assesses hygiene competencies in the areas of technical knowledge and practical skills [23]. The vignettes are used to identify points of hygiene-related learning that must then be listed. Twelve pictures were shown for 30 seconds each during the test, depicting a hygiene problem or a hygienically correct situation. If the situation depicted is correctly identified and described, the participants receive one point, so that a total score of 0 to 12 points is possible. The reported item response theory (IRT) analyses on the HygiKo-SJT show that the test is suitable for assessing hygiene competence and to distinguish between individuals who have different hygiene-related competence levels (for 17 of the 20 items/pictures) [23], [24]. In this context, the competency question is particularly sensitive for the low to intermediate competency range [23]. Infection control competencies can be used by nursing and medical students as well as by professional groups in the healthcare system, and help assess the hygiene competence taught [23]. 

The questionnaires were based on the test by Heiniger et al. prepared and translated into Albanian by an interpreter. The translation took place under the guidance of a linguist and cultural scientist. This pragmatic approach was chosen in order to be able to react to the acute situation. Therefore, the translation's translatability and cultural relevance based on the “Translation guidelines and translation documentation of the European Social Survey (ESS)” were the focus of the translation.

All image vignettes and solutions were checked in cooperation with hygiene experts from the hygiene department of the University Hospital Halle (Saale). The generated image vignettes were transferred to a paper-pencil questionnaire as in the original test. For each vignette, respondents were asked

1. to note whether they saw a problem with applicable hygiene guidelines in the image (yes/no dichotomy) and
2. if so, to describe the problem in a short verbal response.

For each item, the Gartmeier et al. suggested solution was accepted. Correct answers were counted if

1. the respondent recognized a procedural error (a hygiene deficit) in the picture and
2. correctly described the hygiene error.

Answers were not counted if the participants only checked the wrong (unhygienic) action, but the error was not described correctly.

## Results

### Project phase 1

In cooperation with the Kolegji Heimerer, relevant RKI (Robert Koch Institut) recommendations were researched in the first six weeks of the first project phase, and the hospital hygiene SOPs of the University Hospital Halle (Saale) were collated with a focus on COVID-19 patients. Together with a contact person from Kollegji Heimerer, the content of the literature found was adapted to the context in Kosovo. Participants were invited to familiarize themselves with evidence-based theoretical content through reading. From reading the specialist literature, the participants asked themselves questions about the content of the online seminars. Based on this, the schedule for the content of the online seminar series could be designed.

### Project phase 2

As part of the second project phase, 25 nurses and doctors from all state clinics (seven regional hospitals and the University Clinic Pristina) participated in a structured online seminar. Of the 25 participants, twelve completed the pre-test questionnaire and 14 completed the post-test questionnaire. Seven participants answered both the pre- and the post-test. In the pre-test, the participants achieved an average total score of 2.58 points (SD 1.31). In the post-test, an average of 3.50 points was achieved (SD 2.56). Using the two-sample t-test assuming different variances, the p-value (two-tailed) of 0.255 (t(20)=–1.171; p=0.255) indicated the difference between the two means. The change in competence could not be statistically confirmed, but the average point value in the post-test was higher. In a final discussion with the participant group at the end of the last online seminar, the participants gave positive feedback with the desire to continue the online format.

## Discussion and conclusions

The orientation towards the Kern-Zyklus [19] for curriculum development proved to be useful in developing this infection control and hygiene educational programme. It enabled a structured development of the educational programme from problem finding to the evaluation of the results. The initial response from the participants about knowledge transfer as part of this digital education programme was positive. At the end of the course, participants expressed the wish to use this format for further training and education. The translation and communication of SOPs in the course of the first project phase was an effective and necessary measure that can be implemented quickly in order to provide information on work processes, preventive measures in a timely manner, define the main content at an early stage, and address these in the subsequent online seminars.

In the second phase of the project, the video training was transmitted without any problems through the video-conferencing system. Cooperation with the interpreter and the consecutive interpreting process was effective in communicating the content of the programme. The interaction between the interpreter and the speaker improved significantly over the course of the seminar series. In an interactive project and from a linguistic point of view, communication problems can never be completely ruled out, even with the help of native-speaker interpreters. The opportunities for discussion and questions after each seminar were well received by the participants. The interaction between the instructors and participants was also reported to enhance participants’ role of subsequently acting as multipliers for hygiene knowledge. 

A statistically significant difference in the pre-post survey could not be demonstrated. The evaluation by the HygiKo-SJT was made difficult, for example, by the slow return the questionnaires by the participants, although this could be related to their ongoing work in dealing with the COVID-19 pandemic. A before and after measurement makes sense for the evaluation of this project in order to show the direct learning success. However, if the internal validity is low, it is unclear whether learning success can be attributed to the educational concept. In this regard, testing in an experimental design with a control group could be a next step in evaluating this type of educational programme. Furthermore, the test as an instrument is still preliminary and has not yet been sufficiently developed in terms of pre-post application in this context. The very small random sample group in this project may also be a contributing factor. A positive aspect is that the HygiKo-SJT has key advantages in practicability and resource requirements compared to conventional evaluation methods (direct compliance check or restriction to hand hygiene). The test could provide a good evaluation aid in a cross-sectional application in connection with digital education concepts.

Overall, digital hygiene training courses are a suitable option for knowledge transfer for a large number of participants in different regions and institutions in order to impart hygiene knowledge and practical skills. The possibility for direct exchange during the interactive online training enabled questions and discussions. Participants were able to react to locally existing hygiene problems (e.g., the lack of personal protective equipment) and to develop individual solutions and recommendations for action. The individual sessions could also be adapted to the prevailing situation in the Republic of Kosovo. The participants reported that they were able to transfer the theoretical and practical training content directly into their everyday clinical work. Furthermore, the question arises whether a similar training model in longitudinal studies would contribute to sustainable training of hygiene knowledge and anchoring the hygiene competence of the health professions. More extensive work could address the question of whether similar training concepts could generally contribute to improving hospital hygiene structures, reducing infections acquired in hospitals and thus improving patient safety and the quality of patient care.

This rapidly developed online educational programme shows that in times of crisis, theoretical and practical knowledge of hygiene can be conveyed to nurses and medical staff over long distances; this is of particular interest concerning the “local flexibility” of the participants in the target country. All participants can follow the training courses from different locations and do not have to come together at a central location. This reduces the risk of infection in a pandemic situation and represents an opportunity for organizational effort and feasibility. This method of delivering online education also makes it possible to train a larger group of people who then act as multipliers and pass on hygiene-related knowledge to colleagues. The decision to cooperate was made more accessible for the participating institutions, because the online format of the educational concept made it possible to minimize costs due to absence from work or delays in the work process.

## Notes

### Competing interests

The authors declare that they have no competing interests.

### Funding

This manuscript was created as part of a grant for the program “Special competition COVID-19 Response: Together against the pandemic” (project number: 1210038.105.86) by the German Society for International Cooperation (GIZ).
